# A Care Bundle Aiming to Reduce the Risk of Obstetric Anal Sphincter Injury: A Survey of Women's Experiences

**DOI:** 10.1111/1471-0528.18029

**Published:** 2024-12-11

**Authors:** Magdalena Jurczuk, Lizzie Phillips, Posy Bidwell, Dorian Martinez, Louise Silverton, Nick Sevdalis, Jan van der Meulen, Ipek Gurol‐Urganci, Ranee Thakar

**Affiliations:** ^1^ Centre for Quality Improvement and Clinical Audit Royal College of Obstetricians and Gynaecologists London UK; ^2^ Maternity Services University Hospital Plymouth NHS Trust Plymouth Devon UK; ^3^ South Warwickshire Foundation Trust Warwick UK; ^4^ Royal College of Midwives London UK; ^5^ Centre for Behavioural and Implementation Science Interventions, Yong Loo Lin School of Medicine National University of Singapore Singapore Singapore; ^6^ Department of Health Services Research and Policy London School of Hygiene and Tropical Medicine London UK; ^7^ Obstetrics and Gynaecology Croydon University Hospitals NHS Trust Croydon UK

**Keywords:** childbirth experience, decision‐making, intrapartum care, obstetric anal sphincter injury, perineal tear, women's views

## Abstract

**Objective:**

To study experiences of women who gave birth in maternity units that have implemented a ‘care bundle’ quality improvement initiative to reduce obstetric anal sphincter injury (OASI) and associated morbidity.

**Design:**

Postnatal electronic questionnaire.

**Setting:**

Twenty‐nine maternity units across England, Scotland and Wales.

**Population:**

Women with live vaginal births.

**Methods:**

Descriptive statistics for quantitative results. Analysis of free‐text responses informed by framework method.

**Main Outcome Measures:**

Experience with components of the care bundle: information provision, manual perineal protection (MPP) and post‐birth rectal examination.

**Results:**

In this study, 1208 women completed the survey: 684 (56.6%) said they received antenatal information about perineal health, 377 (31.2%) recalled MPP, and 664 (55.0%) recalled rectal examination. Of the 684 women who said they received antenatal information, 595 (87.0%) agreed that the information was easy to understand, and 423 (61.8%) agreed that it helped them to make their own choices to reduce their OASI risk. One hundred and fifty‐four (22.5%) agreed that the information made them fearful about giving birth vaginally. Of the 377 women who recalled MPP, 203 (53.9%) felt it provided them with support, and another 97 (25.7%) did not mind the sensation. Of the 664 women who recalled rectal examination, 281 (42.3%) did not mind the exam, and another 335 (50.5%) felt it was uncomfortable but understood its importance. Free‐text responses aligned with quantitative results.

**Conclusions:**

Many women did not recall MPP or rectal examination. The reported experiences for those who recalled these components do not support concerns that the OASI care bundle has negative effects on women's experiences.

## 1 Introduction

Approximately 80% of women and birthing people giving birth vaginally experience some degree of perineal trauma, most of which is minor and will heal well [1]. Less frequent but more severe is an obstetric anal sphincter injury (OASI), which can have a significant long‐term impact on a woman's quality of life, because the injury may cause pain and anal incontinence [2, 3, 4] and consequently may lead to social, psychological and emotional difficulties [5, 6].

Increases in OASI rates have been reported in a number of countries [7, 8, 9], which prompted several quality improvement initiatives [10, 11, 12]. One of these initiatives is the OASI care bundle (OASI CB), developed to be implemented in maternity units in the National Health Service (NHS) in England, Scotland and Wales [13]. In a previous study, we showed that this care bundle, which includes recommendations for information that women should receive during pregnancy, manual perineal protection (MPP) during the birthing process, an episiotomy when indicated and a rectal examination after birth reduced OASI rates by 20% without affecting the rates of caesarean birth or episiotomy [14].

A systematic review of studies exploring women's experiences of maternity care highlighted the importance of women being the centre of decision‐making and their ability to choose how and in what way they want to give birth to their baby [15]. This is especially relevant for the recommended MPP and post‐birth rectal examination, which attracted considerable controversy after the OASI CB was published [16, 17].

Using qualitative research, we have already provided some evidence that women did not feel that the OASI CB affected ‘their physical integrity’ [18]. In this paper, we report further evidence based on quantitative findings of a postnatal survey of women's experiences with the OASI CB, with a focus on the information that was received and their experiences of MPP and rectal examination after birth.

## 2 Methods

### 2.1 The OASI2 Study

**FIGURE 1:**
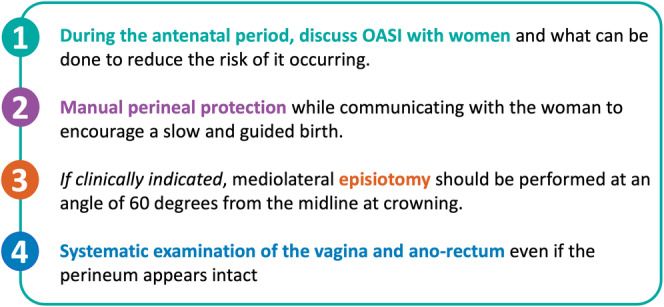
Summary of the OASI care bundle components.

The OASI CB consists of four components, summarised in Figure 1. Following the original study of its clinical effectiveness (OASI1 study) [14], the OASI CB's ‘scalability’ and ‘sustainability’ with different implementation approaches were evaluated in the OASI2 study [19]. All 29 maternity units participating in the OASI2 study received an implementation toolkit of resources to support rolling out and sustaining the OASI CB in each unit, including a clinical manual, and an antenatal discussion guide which included information on perineal massage, use of warm compresses, movement during labour, birth positions, manual perineal protection and the use of an episiotomy when indicated (Supporting Information Data S1). The OASI2 study also included a survey exploring women's experiences during the birthing process. The results of this survey are reported in this paper.

### 2.2 Women's Involvement

Women were closely involved in all components of the OASI1 and OASI2 studies. Two women who sustained an OASI were lay representatives on the Independent Advisory Group (IAG) of the OASI2 study and the project team also worked closely with UK‐based support groups (see Acknowledgements).

### 2.3 Survey Design and Delivery

A 32‐item bespoke survey of women's experiences with the OASI CB was designed by the OASI2 project team in collaboration with the IAG, also taking into account responses following the publication of the OASI1 results [16, 17]. Apart from collecting information about the information that women received and their experiences of MPP and rectal examination after birth, the survey also included a section on their characteristics and intrapartum care. The survey questionnaire was only available in English and had to be completed on an electronic device.

All 29 participating maternity units were sent a printed poster that included information on the purpose of the survey (Supporting Information Data S2). The posters included a link and ‘Quick Response’ (QR) code. Women were informed about the survey by midwives before leaving the maternity unit. Informed consent was implied when a survey was submitted. Survey responses did not include identifying patient details (e.g., name, address or NHS number). Therefore, their responses could be not linked to any clinical data, and it was not possible to send reminders to eligible women who had not completed the survey.

An optional free‐text field was included at the end of the survey for respondents wishing to provide additional detail about their experience.

### 2.4 Study Participants

Women were eligible to be included if they had a vaginal birth during the OASI2 implementation period (between 1 October 2021 and 31 December 2022) in any of the 29 participating maternity units and had given birth less than 6 weeks ago at the time of the survey completion.

### 2.5 Analysis

The age, ethnicity and country in which birth took place as well as relevant aspects of intrapartum care for women who responded were compared with the entire eligible population, defined as those women who were included in the OASI2 study. Age was grouped into four categories (< 25, 25–29, 30–34, ≥ 35 years) and ethnicity into two (‘a White ethnic background’ and ‘all other ethnic backgrounds’). Forceps and ventouse births were combined into one category (‘instrumental birth’). Six items, asking respondents to indicate whether they agree with statements about the information they had received, had a five‐level response category. When analysing these items, we used a dichotomised response (‘strongly agree’ and ‘agree’ versus ‘neither agree nor disagree,’ ‘disagree’ and ‘strongly disagree’).

Frequencies were presented using contingency tables. Chi‐squared test was used to test the statistical significance of differences in percentages and a *p* value smaller than 0.05 was considered to indicate a statistically significant result. Stata 16 software was used for all statistical analyses.

Free‐text responses were analysed with Microsoft Excel by three authors (M.J., D.M. and L.P.) using an approach informed by the framework method [20]. After an initial coding of all responses, specific themes were identified, related to OASI CB components.

## 3 Results

A total of 1506 survey responses were submitted, of which 298 were excluded from the analysis (25 women who gave birth in non‐participating units, 211 women who had a caesarean birth and 62 women who gave birth vaginally but more than 6 weeks ago at the time of survey completion). Responses from 1208 women were eligible for inclusion. Respondents were typically older and more likely to be from a White ethnic background, more likely to have given birth in England, and to have had an instrumental birth, compared to all eligible women giving birth in the 29 participating units in the same period (Table 1). The percentage of respondents who reported having had an OASI (119/1208 [9.9%]) was considerably higher than the OASI rate (1921/69534 [2.8%]) observed in all eligible women.

**TABLE 1:** Characteristics reported by the 1208 survey respondents compared to the eligible population in the 29 participating maternity units.

	Reported by survey respondents; % (*n*)	Recorded in the eligible population; % (*n*)
Number of women	1208	69 534
Age (years)		
< 25	9.9% (120)	16.6% (11375)
25–29	25.6% (309)	27.3% (18708)
30–34	38.5% (465)	34.6% (23720)
35 +	26.0% (314)	21.5% (14727)
*Missing*	*[0]*	*[1.4% (1004)]*
Ethnic background		
White	84.3% (1018)	75.5% (50114)
Asian/Asian British	7.3% (88)	13.1% (8725)
Black/African/Caribbean/Black British	4.1% (50)	5.6% (3745)
Mixed ethnic background	2.6% (31)	2.6% (1749)
Other ethnic background	1.7% (21)	3.1% (2073)
*Missing*	*[0]*	*[4.5% (3128)]*
Country where the birth took place		
England	89.9% (1086)	80.3% (55821)
Scotland	5.6% (68)	10.4% (7217)
Wales	4.5% (54)	9.3% (6496)
Mode of vaginal birth		
Spontaneous vaginal birth	77.2% (927)	80.1% (55696)
Instrumental birth	22.8% (273)	19.9% (13838)
*Missing*	*[0.7% (8)]*	*[0]*
Episiotomy	28.1% (339)	25.5% (17699)
Obstetric anal sphincter injury	9.9% (119)	2.8% (1921)

*Note:* Percentages are given for respondents with available data. The italicized values were not included in the percentage of total distributions in the main; they represent the proportion of missing data.

Six hundred and eighty‐four (56.6%) of the 1208 respondents recalled receiving perineal health information from a clinician (midwife or obstetrician) in the antenatal period (Table 2). This proportion was 58.3% (594/1018) in women from a White ethnic background and 47.4% (90/190) in women from other ethnic backgrounds (*p* = 0.01; Table S1 in Supporting Information Data S4).

**TABLE 2:** Experiences with the OASI‐CB recalled by the 1208 respondents.

	All respondents; *n* = 1208
Component 1: antenatal discussion	
Having received information from a clinician about perineal tears	56.6% (684)
Component 2: manual perineal protection	
Clinician explaining the benefit of hands supporting the perineum	44.7% (540)
Feeling hands supporting the perineum during birth	31.2% (377)
Component 3: mediolateral episiotomy	
Having an episiotomy	28.1% (339)
Component 4: post‐birth examination	
Recalls clinician explaining the benefit of a rectal examination after birth	48.8% (589)
Clinician performing a rectal examination after birth	55.0% (664)

Of the 684 women who recalled receiving perineal health information, 486 (71.1%) received this information at an appropriate time (after 28 weeks of gestation but before the onset of labour), and only 109 (15.9%) had a verbal discussion with their clinician *and* received the leaflet (Table 3). Five hundred and ninety‐five (87.0%) indicated that the information was easy to understand, 417 (61.0%) that the information helped them to understand the possible long‐term consequences of severe perineal tearing, 487 (71.2%) that the information was sufficient to give or withhold informed consent, and 423 (61.8%) that they felt empowered to make choices to reduce their risk of tearing. However, 154 (22.5%) agreed that the information made them fearful of having a vaginal birth. Women who had a verbal discussion with their clinicians as well as receiving the leaflet felt more positively about information provision than those who did not (Table S2 in Supporting Information Data S4).

**TABLE 3:** Antenatal information according to the 684 women who recalled receiving information from a clinician.

	All women; *n* = 684
Timing and mode of information provision	
The information was received after 28 weeks and before the onset of labour	71.1% (486)
The information was provided with a verbal discussion with clinician *and* the leaflet	15.9% (109)
‘Agree’ or ‘strongly agree’ with the following:	
The information was easy to understand	87.0% (595)
The information helped me understand the possible long‐term consequences of severe perineal tearing	61.0% (417)
The information made me feel empowered to make choices to reduce my risk of perineal tearing	61.8% (423)
The information made me fearful of giving birth vaginally	22.5% (154)
The information was sufficient for me to give or withhold my informed consent	71.2% (487)

*Note:* These results represent collapsed responses derived from a five‐point scale: ‘strongly agree’, ‘agree’, ‘neither agree nor disagree’, ‘disagree’ and ‘strongly disagree’.

Antenatal information was mentioned by 30 respondents in free‐text responses. The key themes were that no or insufficient information was received, that the information was received only after the respondent explicitly asked for it, or the satisfaction with the information received (see Box in Supporting Information Data S4).

Five hundred and forty (44.7%) of the 1208 respondents reported that a clinician had explained the benefits of MPP (Table 2). Three hundred and seventy‐seven (31.2%) recalled feeling hands supporting the perineum (i.e., MPP) during the birthing process. Corresponding percentages of recalling MPP were 36.4% (337/927) in women who had a spontaneous vaginal birth, compared to 13.9% (38/273) in those who had an instrumental birth (*p* < 0.001); and 35.1% (303/864) in those who did not have an epidural, compared 21.2% (72/339) in those who had an epidural (*p* < 0.001; Table S1 in Supporting Information Data S4).

**FIGURE 2:**
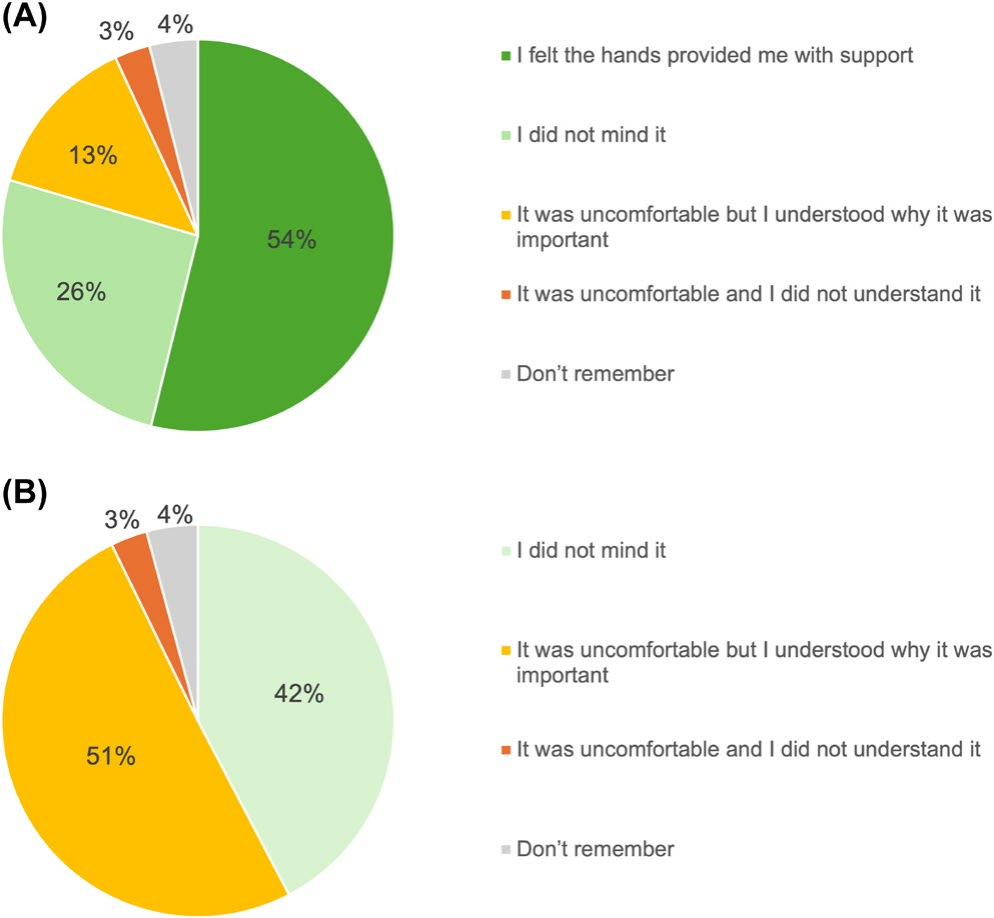
(A) Responses from the 377 women who recalled receiving manual perineal protection. (B) Responses from the 664 women who recalled a rectal examination.

Of the 377 women who recalled receiving MPP, 203 (53.9%) felt that it provided them with support, 97 (25.7%) did not mind the sensation, and 51 (13.5%) felt it was uncomfortable but understood its importance (Figure 2A).

There were 17 mentions of ‘hands on’ or ‘perineal support’ in free‐text responses. These were focused on positive experiences with receiving this practice or disappointment over not receiving it despite requesting it (see Box in Supporting Information Data S4).

Five hundred eighty‐nine (48.8%) of the 1208 respondents reported that a clinician had explained the benefits of a rectal examination (Table 2), corresponding percentages were 50.9% (514/1018) in women from a White ethnic background, compared to 39.5% (75/190) in women from other ethnic backgrounds (*p* = 0.02). Six hundred sixty‐four (55.0%) recalled having a rectal examination after birth. The percentage of women recalling a rectal examination was 56.9% (579/1018) in women from a White ethnic background, compared to 44.7% (85/190) in women from other ethnic backgrounds (*p* = 0.002) (Table S1 in Supporting Information Data S4). Corresponding percentages were 61.0% (565/927) in women who had a spontaneous vaginal birth, compared to 34.4% (94/273) in those who had an instrumental birth (*p* < 0.001); and 60.1% (519/864) in those who did not have an epidural, compared to 40.9% (142/339) in those had an epidural (*p* < 0.001; Table S1 in Supporting Information Data S4).

Of the women who recalled a rectal examination after birth, 281 (42.3%) did not mind the examination, a further 335 (50.5%) felt it was uncomfortable but understood its importance, and 20 (3.0%) did not understand the purpose of the practice (Figure 2B).

Post‐birth rectal examination was mentioned in 14 free‐text responses. These responses highlighted either good communication with the clinician and feeling well informed about the intervention or feeling surprised by the examination and not having sufficient information about it (see Box in Supporting Information Data S4).

## 4 Discussion

### 4.1 Main Findings

There is a gap in the evidence base regarding how improvement interventions influence women's childbirth experiences. To the best of our knowledge, this is the largest study of women's experiences giving birth in maternity units that have implemented a care bundle recommending MPP and systematic vaginal and rectal examination after birth to reduce the risk of OASI and associated morbidity.

About one in two responding women recalled receiving information about perineal health and this information was not always provided at an appropriate time nor in the recommended format (leaflet and verbal discussion with clinician). Only about one in three of the respondents recalled receiving MPP and four in five of these women indicated that they did not mind the sensation of hands touching their perineum or that it was uncomfortable but understood its importance. About one in two recalled a post‐birth rectal examination and over nine in 10 of these women indicated that they did not mind this examination or that it was uncomfortable but understood its importance. Taken together, this suggests either that these components were not carried out or that they were carried out but not remembered. Lastly, for most women who recalled these components, reported experiences were positive.

### 4.2 Strengths and Limitations

All survey materials developed for the OASI2 study, particularly the antenatal discussion guide leaflet and survey, of which the results are reported in this paper, were designed together with women who had given birth in NHS maternity units taking part in the study, and women who had experienced an OASI. This, along with lessons learned from our previous process evaluations of OASI CB implementation [18], ensured that the survey asked questions about what matters most to women within the context of the OASI CB. The survey's free text question also captured women's narratives which overall supported the quantitative findings.

This study has a number of limitations. First, while there were over 1000 responses to the survey, this represents only a small proportion of the eligible population giving birth in the OASI2 maternity units, with some evidence that older and White women as well as those having had an instrumental delivery were more likely to respond. Therefore, we cannot exclude a selective response which restricts the representativeness and generalisability of the findings compared to surveys with probabilistic samples [21, 22]. For example, the OASI rate among the respondents is considerably higher than the corresponding rate in the entire eligible population in the OASI2 units. This demonstrates that, if there is a bias due to selective participation, women who responded to the survey were more likely to have had a negative experience than the women overall in the eligible population who gave birth in the 29 maternity units participating in the OASI2 study. Second, we also need to accept that the survey responses may be inaccurate. However, because the responses come from the women themselves, they should be considered accounts from interested and impartial observers.

### 4.3 Interpretation (in Light of Other Evidence)

The percentage of respondents who recalled receiving perineal health information, MPP, or a rectal examination was lower than expected. There are three possible explanations: the OASI CB component was not offered by clinicians, the component was offered to women, but it was not accepted, or the component was offered and provided but women did not recall it at the time of the survey.

There is some evidence that all these are possible and relevant explanations for the results of our study. First, low recall of antenatal information can be explained by ‘information overload’ often experienced by pregnant women, making it difficult to remember specific content [23]. Second, the reliability of recall of intrapartum practices is uncertain. Large‐scale studies comparing maternal recall with clinical data suggest that recall is generally considered reliable for birth outcomes such as mode of birth [24] and measures associated with the baby, such as birthweight or gestational age [25]. Third, evidence on maternal recall on more specific sensations, like feeling hands on the perineum or whether a rectal examination was done in the moments after birth, was only available from our previous qualitative study: for both MPP and rectal examinations, 5 out of 19 interviewed women who had both components did not recall them [18].

Some clinicians have indicated they feel that providing antenatal information about OASI is ‘too scary’ or ‘too explicit’ for women [26]. Also, concerns have been raised that MPP restricts birth position [27] and that a post‐birth examination may cause distress to women [16]. An Australian qualitative study concluded that the focus on compliance with the care bundle had detrimental effects on supporting women's decision‐making autonomy [28]. However, the results of our survey demonstrate that the experiences with a care bundle aiming to reduce the risk of severe perineal trauma are likely to be positive overall, provided that the materials supporting its implementation reinforce the importance of good communication and support for informed decision‐making and birth choices [29].

Our results reveal three specific areas of improvement. First, while most women felt positive about the information they had received, this was not true for all. This is in line with other studies that indicated that ‘not being listened to’ or ‘consulted with’ is a recurring theme in recent investigations of women's experiences of maternity care [30]. Second, we also found that many women do not recall having received antenatal information. This is in line with the results of the most national survey of women who had given birth in English NHS maternity units, which demonstrate that two in five women were not always given the information and explanations they needed and one in four women were not always involved in decisions about their care during labour and birth [31]. Third, we found that fewer women from a minority ethnic background recalled receiving antenatal information about perineal health, an explanation about post‐birth rectal examination, or the examination itself when compared to white women. Persistent inequalities between ethnic and socioeconomic groups in NHS maternity care [32, 33, 34, 35] highlight the importance of tailoring care and communications to address the needs of pregnant women from diverse backgrounds [36, 37].

## 5 Conclusion

The results of this survey do not support concerns that the implementation of a care bundle to reduce the OASI risk has a negative effect on the experiences of women giving birth. It is important to note that many women did not recall that they had perineal health information, MPP or a post‐birth rectal examination. This demonstrates that further implementation efforts are needed to ensure that the OASI CB, which reduces the risk of severe perineal trauma by 20% [14], is implemented as intended.

## Author Contributions

R.T., L.S., P.B., I.G.‐U., N.S. and J.v.d.M. obtained funding. The study was conceived and designed by all authors. M.J., D.M. and L.P. performed the analyses. R.T., P.B., L.S., D.M., N.S., I.G.‐U. and J.v.d.M. assisted with the interpretation of results. M.J. wrote the manuscript, with input from all other authors. The joint senior authors have made an equal contribution to this study and manuscript. All authors approved the final manuscript.

## Ethics Statement

The OASI2 study received full ethics approval from the Social Care Research Ethics Committee on 1 December 2020 (REC reference 20/IEC08/0029). This ethical opinion covers all aspects of the research and is valid across all participating sites and received ethics approval from the Health Research Authority (HRA). OASI2 is registered on both the National Institute for Health Research (NIHR) Clinical Research Network (CRN) Portfolio and ISRCTN registry (ISRCTN26523605).

## Conflicts of Interest

N.S. is the director of London Safety and Training Solutions Ltd., which offers training in patient safety, implementation solutions and human factors to healthcare organisations and the pharmaceutical industry. R.T. is the immediate past president of the International Urogynecological Association (IUGA) and is currently the president of the Royal College of Obstetricians and Gynaecologists (RCOG). The other authors have no competing interests to declare.

## Supporting information


Data S1.

Data S2.

Data S3.

Data S4.

## Data Availability

Research data are not shared.
