# Supplementary material for: A Care Bundle Aiming to Reduce the Risk of Obstetric Anal Sphincter Injury: A Survey of Women's Experiences
Source: BJOG. 2024 Dec 11;132(5):588–95. doi: 10.1111/1471-0528.18029 (PMC11879911; doi:10.1111/1471-0528.18029)
Supplement: Supplementary file 1 — Data S1. [file BJO-132-588-s004.pdf]

# Perineal Health
## in Pregnancy, Birth & Beyond
Antenatal discussion guide

OASI TWO CARE BUNDLE

V5 AUGUST 2023

## What types of perineal tears can occur during childbirth?

During vaginal birth, it is very common to experience a graze or tear of the labia or, more frequently, the **perineum**, which is the **area between your vagina and anus**. For most, these tears are minor and heal quickly.

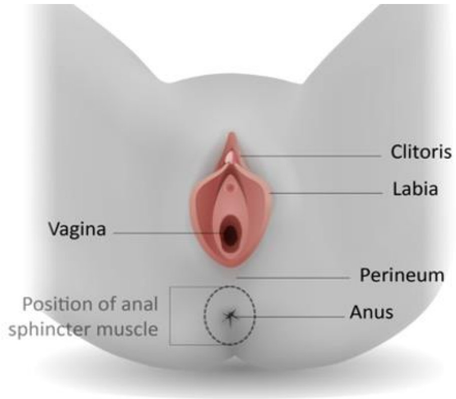


- **1st and 2nd degree:** tears that involve muscle/skin that may require stitches
- **3rd and 4th degree:** severe tears which extend to the muscles that control the anus (the anal sphincter), requiring stitches. **These are also called 'Obstetric Anal Sphincter Injuries' (OASI).**

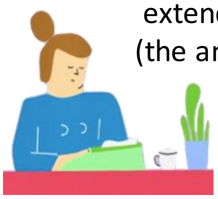

## Am I at risk of a severe tear?

Severe tears (OASI) occur in **3-4 in 100 births.** You are at significantly higher risk if:

- Forceps are used to help you give birth
- This is your first vaginal birth

The following may also increase your risk:

- You sustained a previous OASI
- Your baby is born in the back-to-back position
- Your baby is over 4kg (9 lbs)
- You are of South Asian ethnicity
- Your baby is born quickly
- You are over 35 years of age
- Your baby's shoulder gets stuck behind the pubic bone (shoulder dystocia)
- Ventouse is used to help you give birth
- The pushing phase of labour takes a long time

The alternative to a vaginal birth is a caesarean birth. Caesarean birth has different risks to yourself, your baby and your future pregnancies.

## What about recovery?

Most women and birthing people who have a severe tear (OASI) repaired recover well, although it can take some time. Occasionally, long-term pain and a difficulty or inability to fully control the bladder, bowels or the passing of wind can occur. This could lead to:

- Feelings of depression, low mood, isolation
- Anxiety about leaving the house and not being able to quickly access a toilet
- Difficulty bonding with your baby
- Concerns about leakage while exercising
- Concerns about having sex or giving birth again

**If you experience any of the above after birth,** contact your doctor or midwife as soon as possible to access specialist care.

**BRAIN can help you ask questions**

The BRAIN acronym helps you have conversations that will support you to make a decision.

**B** = what are the benefits?
**R** = what are the risks?
**A** = what are the alternatives?
**I** = what does your intuition tell you?
**N** = what happens if we do nothing for now?

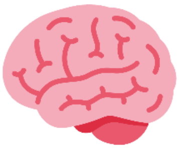

Royal College of Midwives
Royal College of Obstetricians & Gynaecologists
The Health Foundation

# How can I reduce my risk of a severe tear?

**Perineal massage** with a natural oil (such as coconut or almond) from 35 weeks pregnant until birth, as illustrated here. Visit rcog.org.uk/tears for more information on how to do this.

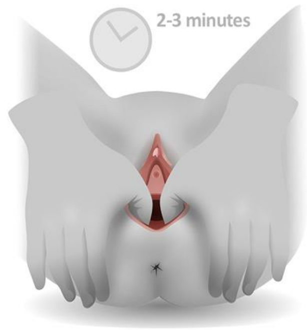


**A warm compress** is a flannel heated with warm tap water and held against your perineum during the pushing phase of labour. Ask your midwife or doctor if they can provide this.

**Spontaneous vaginal birth** *(birth without forceps or ventouse)* can be encouraged by choosing the ideal place of birth (consider a homebirth or midwifery-led unit if you are low- risk), avoiding induction and epidural where possible, creating a relaxing environment (consider soothing lighting, sounds, smells) and remaining active throughout labour and birth. *For first-time mothers with an epidural,* lying on your side during the pushing phase of labour is recommended.

**Choose a birth position that is most comfortable for you.** Listen to your midwife and they will advise a slow and guided birth of the head. Positions at the moment of birth that may reduce risk of severe tears include:

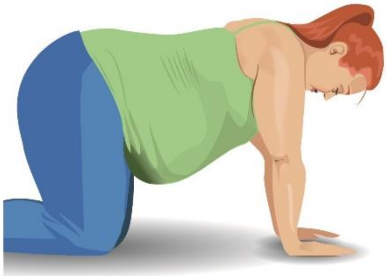

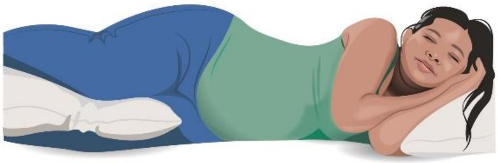

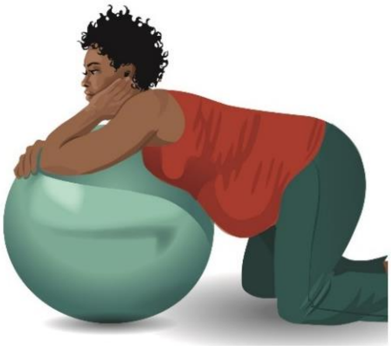

The **OASI Care Bundle** is the following set of practices, most effective when applied together:

1. In the antenatal period, your midwife or doctor will **discuss severe tears (OASI) with you** and what can be done to reduce the risk of this occurring.
2. With your consent, your midwife or doctor will **use their hands to support** your perineum and the baby's head and shoulders during birth and encourage a slow and guided birth.
3. You may need an **episiotomy**—a cut through the vaginal wall and perineum to make more space for your baby to come out—your midwife or doctor will ask for your consent to do this.
4. After your baby has been born, your midwife or doctor will ask for your consent to **examine your vagina, perineum and anus** (just inside the back passage) to ensure any tears are identified and appropriately treated to avoid further consequences.

✓ **Developed by experts**

✓ **Supported by women**

✓ **Found effective in a 2017-18 study (OASI1)**

**Please speak to your midwife or doctor if you have any questions.**
**For more information and support, visit: rcog.org.uk/tears or masic.org.uk**

Royal College of Midwives

Royal College of Obstetricians & Gynaecologists

The Health Foundation
