# Supplementary material for: A Care Bundle Aiming to Reduce the Risk of Obstetric Anal Sphincter Injury: A Survey of Women's Experiences
Source: BJOG. 2024 Dec 11;132(5):588–95. doi: 10.1111/1471-0528.18029 (PMC11879911; doi:10.1111/1471-0528.18029)
Supplement: Supplementary file 2 — Data S2. [file BJO-132-588-s002.pdf]

Postnatal Survey

The OASI Care Bundle study team appreciates you taking the time to complete this survey, in which we ask you to reflect on the care you received throughout your pregnancy and during your labour and birth, particularly related to perineal trauma. The perineum is the area between the vaginal opening and the rectum (just inside the back passage). Your responses to the survey will contribute to the development of recommendations to improve aspects of maternity care related to perineal tears.

The survey is predominantly multiple choice and we will not be able to trace your answers back to you. Please also ensure that you do not disclose any personal information in your free text responses that could identify either yourself, your healthcare providers (doctors, nurses, midwives, etc.) or anyone else.

This survey has four parts. Part A asks some general questions about you, Part B asks about any information you received about perineal tears, Part C asks about your labour and birth experience and Part D is about the period right after your baby's birth. Please make any additional comments in the final text box.

Although we do not intend for this survey to upset you, we acknowledge that some questions may bring up memories or feelings of discomfort experienced during labour or birth. At the end of this survey, we provide a list of resources that may provide you with additional support.

Thank you very much for taking this survey.

Eligibility:

1. Did you have a vaginal birth in the last four weeks (not caesarean birth)?
   a. Yes
   b. No→ Thank you for your interest in completing the survey.

2. In which Trust did you most recently give birth?
   a. [Trust name drop down menu.]
   b. None of the above → Thank you for your interest to completing the survey.

About you:

1. How long ago did you give birth? Consider your most recent birth.
   a. < 48 hours ago
   b. 1-2 weeks ago
   c. 3-4 weeks ago
   d. 5-6 weeks ago
   e. > 6 weeks ago
2. Age (drop down menu with age groupings)
   a. <20
   b. 20-24
   c. 25-29
   d. 30-34
   e. 35-40
   f. >40
3. Ethnicity (drop down menu with options)
   a. White
   b. Mixed ethnicity
   c. Asian/ Asian British
   d. Black/ African/ Caribbean/ Black British
   e. Other ethnic group
4. Did you have a severe perineal tear (known as a third or fourth degree tear)?
   a. Yes, during this birth
   b. Yes, in a previous birth
   c. No
   d. Don't know
5. Do you know anyone who has had a severe perineal tear?
   a. Yes
   b. No
   c. Don't know
6. After your most recent birth, have you leaked poo from your back passage?
   a. Yes
   b. No

A. Information about tears searched for or received prior to birth of baby:

7. Did you receive any of the following information from your midwife/doctor about perineal tears and ways to prevent them in antenatal care? [check all that apply]
    a. Yes, my midwife/doctor gave me a leaflet (no verbal explanation/discussion) in antenatal care.

    b. Yes, my midwife/doctor discussed perineal tears and prevention with me in antenatal care.

    c. I received information from my midwife/doctor in antenatal care through other means
        i. Please specify: __________(free text)

    d. No, I did not receive any information about perineal tears during antenatal care.

8. Did you receive any of the following information from your midwife/doctor about tearing during childbirth and ways to prevent them in hospital, prior to birth?
    a. Yes, my midwife/doctor gave me a leaflet (no verbal explanation/ discussion) in hospital, prior to birth.

    b. Yes, my midwife/doctor explained perineal tears and prevention with me in hospital, prior to birth.

    c. I received information from my midwife/doctor in hospital, prior to birth through other means
        i. Please specify: __________(free text)

    d. No, I did not receive any information about perineal tears in hospital prior to birth.

9. For each statement, please select an answer that best suits your experience with receiving information about perineal tears and ways to prevent them from your midwife/doctor ***(skip to question #8 if you did not receive this information from your midwife/doctor):***
    a. *The information was easy to understand.*

5	4	3	2	1
Strongly agree	Agree	Neither agree nor disagree	Disagree	Strongly disagree

    b. *The information helped me understand the possible long term consequences of severe perineal tearing.*

5	4	3	2	1
Strongly agree	Agree	Neither agree nor disagree	Disagree	Strongly disagree

    c. *The information made me fearful of giving birth vaginally.*

5	4	3	2	1
Strongly agree	Agree	Neither agree nor disagree	Disagree	Strongly disagree

d. *The information made me feel empowered to make choices to reduce my risk of perineal tearing.*

5	4	3	2	1
Strongly agree	Agree	Neither agree nor disagree	Disagree	Strongly disagree

e. *I felt encouraged to ask questions about the information.*

5	4	3	2	1
Strongly agree	Agree	Neither agree nor disagree	Disagree	Strongly disagree

f. *I felt that the information about possible interventions was sufficient for me to give or withhold my informed consent.*

5	4	3	2	1
Strongly agree	Agree	Neither agree nor disagree	Disagree	Strongly disagree

10. Did you independently search for/receive any information about perineal tears? Please tick all that apply.
    a. A relative or friend told me about tears
    b. I searched for more information from books and magazines
    c. I searched for more information online and/or via an app
    d. I did not search for or receive any additional information on perineal tears.

B. Labour and birth experience:

11. Were you able/ supported to move freely throughout your labour?
    a. Yes ***(skip to #11)***
    b. No

12. Why were you unable/ not supported to move freely throughout labour? Please choose the option that most accurately describes your experience.
    a. I did not know that I had the option to move
    b. The anaesthetic I was given for pain relief did not support me to move
    c. I was continually monitored throughout labour
    d. Other: __________________(free text)

13. Did you give birth in water?
    a. Yes
    b. No

14. How did you choose your birth position? Please choose the option that most accurately describes your experience.
    a. I chose my birth position and my midwife/doctor supported me.
    b. I had to insist on my chosen birth position because my midwife/doctor advised against it.

c. I wanted a different birth position, but I did what my midwife/ doctor recommended.
d. I did not have a preferred birth position; I did what my midwife/doctor suggested.

15. Did you have an epidural? An epidural is an anaesthetic injection into the space around the nerves in your back to numb the lower body.
    a. Yes
    b. No
    c. Don't know/ don't remember

16. Did you have an episiotomy? An episiotomy is a cut made through the vaginal wall and perineum to make more space to deliver the baby.
    a. Yes
    b. No
    c. Don't know/ don't remember

17. What kind of vaginal birth did you have?
    a. I pushed my baby out myself (spontaneous)
    b. The doctor used a suction cup to help guide my baby out (ventouse or kiwi)
    c. The doctor used forceps to help guide my baby out
    d. Don't know/don't remember

18. Did your midwife/doctor explain the benefit of supporting your perineum with their hands during the birth of your baby?
    a. Yes
    b. No
    c. Don't know/don't remember

19. Did you feel hands on your perineum during the birth of your baby?
    a. Yes
    b. No ***(skip to #19)***
    c. Don't know/don't remember ***(skip to #19)***

20. How did you feel about experiencing hands on your perineum during your birth? Please choose the option that most accurately describes your experience.
    a. It was uncomfortable and I did not understand it
    b. It was uncomfortable but I understood why it was important
    c. I did not mind it
    d. I felt that the hands provided me with support
    e. Don't remember

C. Postpartum experience:

21. Did your midwife/doctor explain the benefit of a rectal (back passage) examination after you gave birth?
    a. Yes

b. No
c. Don't know/don't remember

22. Did your midwife/doctor perform a rectal examination with your consent by inserting a finger into your rectum after you gave birth?
    a. Yes
    b. No ***(skip to #22)***
    c. Don't know/ don't remember ***(skip to #22)***

23. How did you feel about the rectal examination? Please choose the option that most accurately describes your experience.
    a. It was uncomfortable and I did not understand it
    b. It was uncomfortable but I understood why it was important
    c. I did not mind it
    d. Don't remember

24. Did your midwife/doctor communicate with you during and after birth about what was happening?
    a. Yes
    b. No

25. Did you feel supported to provide (verbal) **informed consent** to any interventions recommended by your midwife/doctor during and after birth?
    a. Yes
    b. No
    c. Not sure

26. Please feel free to make any additional comments about your birth experience (including birth position, interventions, consent) and the information your received or wish you had received prior to giving birth.

[free text response]

Please talk to a healthcare professional if:

- You are concerned about your physical or mental wellbeing

- You are experiencing ongoing pain or difficulty controlling your bladder and/or bowels six weeks after birth

- Completing this survey has caused you any emotional distress

Additional resources:

- The RCOG Tears Hub contains a series of information sheets for women related to perineal tearing, episiotomy and postpartum recovery. Follow the link for more information.
- **The Birth Trauma Association** is an organisation that supports parents who have been through a traumatic birth experience. Visit the 'Information for parents' page on their website for more information. You may also sign up for their private support group on Facebook.
- **MASIC** is a dedicated charity for women who have experienced anal sphincter injuries. Visit their webpage and if you'd like, sign up for their private support group on Facebook.
- **Patient Advice Liaison Service (PALS):** If you have any concerns about the care you received during your labour and/or childbirth, please contact your Trust's PALS as soon as possible. Follow this link to find the PALS nearest you: https://www.nhs.uk/service-search/other-services/Patient-advice-and-liaison-services-(PALS)/LocationSearch/363
  In Scotland, please refer to the **Patient Advice & Support Service (PASS)**: https://www.cas.org.uk/pass
