# Supplementary material for: A Care Bundle Aiming to Reduce the Risk of Obstetric Anal Sphincter Injury: A Survey of Women's Experiences
Source: BJOG. 2024 Dec 11;132(5):588–95. doi: 10.1111/1471-0528.18029 (PMC11879911; doi:10.1111/1471-0528.18029)
Supplement: Supplementary file 3 — Data S3. [file BJO-132-588-s003.pdf]

OASI TWO
CARE BUNDLE

Tell us about your birth experience!

Your maternity unit is implementing the OASI Care Bundle.
Scan the QR code to tell us how this affected you.

What is the OASI Care Bundle?

Obstetric anal sphincter injury (OASI) is the combined term for severe perineal tears that can occur during childbirth. The perineum is the area between the vaginal opening and the rectum (back passage). Severe tears can have a long-term impact on women's wellbeing. For more information, ask your midwife or doctor for the **'Tears during Childbirth: antenatal discussion guide'**.

Your maternity unit has adopted the **OASI Care Bundle,** which is a set of four practices that have been shown to be effective in reducing risk of severe tears if applied together. For more information on the OASI Care Bundle, please visit: www.rcog.org.uk/OASICareBundle

Why complete this survey?

It is important to us that you were supported to have an individual birth plan and a positive birth experience. This is an opportunity to share your experience and help us understand how the OASI Care Bundle could be improved. **This survey takes 10-15 minutes to complete. The information gathered is anonymous and will be securely stored. You may complete the survey up to 6 weeks after giving birth.**

You may also access the survey through this webpage:

https://bit.ly/3DzAGsl

Royal College of Obstetricians & Gynaecologists | The Health Foundation | NHS Croydon Health Services NHS Trust | London School of Hygiene & Tropical Medicine | Royal College of Midwives

Version 2.0/ 19.11.2021
