# Supplementary material for: A Care Bundle Aiming to Reduce the Risk of Obstetric Anal Sphincter Injury: A Survey of Women's Experiences
Source: BJOG. 2024 Dec 11;132(5):588–95. doi: 10.1111/1471-0528.18029 (PMC11879911; doi:10.1111/1471-0528.18029)
Supplement: Supplementary file 4 — Data S4. [file BJO-132-588-s001.docx]

**SUPPLEMENTARY INFORMATION**

**A care bundle aiming to reduce the risk of obstetric anal sphincter injury: a survey of women’s experiences**

Magdalena Jurczuk et al.

Centre for Quality Improvement and Clinical Audit,

Royal College of Obstetricians and Gynaecologists,

10-18 Union Street, London, SE1 1SZ, UK

Corresponding author:

Dr Ipek Gurol-Urganci

Department of Health Services Research and Policy

London School of Hygiene and Tropical Medicine

15-17 Tavistock Place, London, WC1H 9SH, UK

**CONTENT**

|  | Page |
| --- | --- |
| **1 Supplementary Tables**   - Table S1: Recall and information provision for OASI CB components, reported by respondent characteristics. - Table S2: Experiences with antenatal information, by how it was received | 2-3 |
| **2 Box**: Free-text responses | 4-5 |
| **3 OASI2 Antenatal discussion guide**: **Perineal Health in Pregnancy, Birth & Beyond** | Separate document |
| **4 OASI2 Postnatal survey poster** | Separate document |
| **5 OASI2 Postnatal survey** | Separate document |

**1 SUPPLEMENTARY TABLES**

**Table S1. Recall and information provision for OASI CB components, reported by respondent characteristics.**

|  | **All women** | **Age** | | | | | **Ethnicity** | | | | **Mode of birth** | | | **Use of epidural** | | |
| --- | --- | --- | --- | --- | --- | --- | --- | --- | --- | --- | --- | --- | --- | --- | --- | --- |
|  |  | ***<25*** | ***25-29*** | ***30-34*** | ***35+*** | **p-value** | ***White*** | ***Other ethnic background*** | **p-value** | ***SVB*** | | ***Instrumental*** | **p-value** | ***No epidural*** | ***Epidural*** | **p-value** |
| N | 1208 | 120 | 309 | 465 | 314 |  | 1018 | 190 |  | 927 | | 273 |  | 864 | 339 |  |
| Component 1: Antenatal information  Received information from clinician about perineal tears | 56.6%  (684) | 59.2% (71) | 60.5% (187) | 55.3% (257) | 53.8% (169) | 0.31 | 58.3%  (594) | 47.4%  (90) | 0.01 | 56.1%  (520) | | 58.6%  (160) | 0.46 | 60.8% (206) | 55.2% (477) | 0.080 |
| Component 2: Manual perineal protection  Recalls clinician explaining the benefit of hands supporting the perineum | 44.7%  (540) | 48.3% (58) | 47.2% (126) | 46.2% (215) | 38.5% (121) | 0.04 | 45.6% (464) | 40.0%  (76) | 0.18 | 47.2%  (438) | | 36.3%  (99) | 0.01 | 49.8% (169) | 42.8% (370) | 0.03 |
| Recalls feeling hands supporting the perineum during birth | 31.2%  (377) | 31.7% (38) | 30.7% (95) | 34.8% (162) | 26.1% (82) | 0.12 | 30.8% (314) | 33.2%  (63) | 0.32 | 36.3%  (337) | | 13.9%  (38) | <0.001 | 35.1% (303) | 21.2% (72) | <0.001 |
| Component 3: Mediolateral episiotomy  Recalls having an episiotomy | 28.1%  (339) | 33.3% (40) | 27.5% (85) | 29.7% (138) | 24.2% (76) | 0.20 | 27.7% (282) | 30.0%  (57) | 0.52 | 9.6%  (89) | | 90.1%  (246) | <0.001 | 57.5% (195) | 16.3% (141) | <0.001 |
| Component 4: Post-birth examination  Recalls clinician explaining the benefit of a rectal examination after birth | 48.8%  (589) | 45.83  (55) | 53.4% (165) | 47.1% (219) | 47.8% (150) | 0.03 | 50.5%  (514) | 39.5%  (75) | 0.02 | 54.6%  (506) | | 29.3%  (80) | <0.001 | 37.2% (126) | 53.5% (462) | <0.001 |
| Recalls the clinician performing a rectal examination after birth | 55.0%  (664) | 52.5% (63) | 60.0% (185) | 54.0% (251) | 52.5% (165) | 0.28 | 56.9%  (579) | 44.7%  (85) | 0.002 | 61.0%  (565) | | 34.4%  (94) | <0.001 | 41.9% (142) | 60.1% (519) | <0.001 |

**Table S2: Experiences with antenatal information, by how it was received**

| **‘Agree’ or ‘strongly agree’ with the following:** | **Discussion with clinician and leaflet**  **n=109** | **Discussion only/leaflet only**  **N=575** |
| --- | --- | --- |
| The information was easy to understand | 92.7% (101) | 85.9% (494) |
| The information helped me understand the possible long-term consequences of severe perineal tearing | 78.0% (85) | 57.7% (332) |
| The information made me feel empowered to make choices to reduce my risk of perineal tearing | 74.3% (81) | 59.5% (342) |
| The information made me fearful of giving birth vaginally | 22.0% (24) | 22.6% (130) |
| The information was sufficient for me to give or withhold my informed consent | 88.1% (96) | 68.0% (391) |

**2 BOX**

**Free-text responses**

| **Antenatal information**  One respondent who did not receive this information shared:  *I am shocked completing this survey that my community midwife could have provided me with information on how to prevent tearing and I never received this nor did I receive support to my perineum during birth.* (Between 25-29 years old, white, SVB, no epidural)  Another respondent with a previous severe tear illustrated how this discussion supported her to make informed choices even when the clinician recommended a different course of action:  *I previously experienced a 3C tear and was advised at 37 weeks in this pregnancy to have an elective section. This was not what I wanted, and I strongly felt that factors in my first birth significantly impacted upon the tear that I experienced. I felt that with measures through the OASI care bundle, I would be able to […] have a safe vaginal delivery. I was supported to have a discussion with a specialist consultant who explained the risks and benefits of the different options. I felt empowered to make my own decision but did experience some pressure whilst in hospital from other doctors to have a section.* (Between 30-34 years old, white, SVB, no epidural)  **Manual perineal protection**  One respondent shared her positive experience:  *I had a warm compress and perineal pressure applied during pushing along with gentle reminders and breathing down and out. I ended up propped up on my back as I'd just had an exam and didn't have time to turn round onto my knees or feel comfortable to change position once pushing started. Ended with a 2nd degree tear which was repaired nicely by my amazing midwife and her student.* (Between 30-34 years old, white, SVB, no epidural)  Multiple respondents felt strongly about wanting MPP, in one case attempting it themselves and in another having to insist on it to their provider:  *Baby arrived very quickly at home (unplanned) with only a paramedic in attendance. However, I tried to use my own hands to support perineum based on info provided to me by midwife.* (Between 30-34 years old, white, SVB, no epidural)  *I had to insist on perineal protection. I was told by the midwife she would not do it. She did do it, but after much insisting. I was told I needed to deliver on my back, but did not want to as I know this increases the risk of tears […] I felt that I was having to fight to have my perineum protected and given a previous 3rd degree tear, it wasn't the care I wanted or had been reassured I would have in labour.* (Between 30-34 years old, white, SVB, no epidural)  Another respondent’s reflections illustrate that some providers require additional training regarding MPP in different birth positions in order to fully support women’s choices:  *I chose to give birth on all fours. My midwife explained she couldn't protect my perineum as well in that position compared to on my back. I tried on my back but found it difficult to push. Fortunately, I didn't have any tears or need stitches.* (Between 30-34 years old, white, SVB, no epidural)  **Post-birth examination**  One respondent reflected on feeling reassured after birth thanks to continuous communication from providers:  “*Information received during pregnancy and after birth was very helpful and I felt supported… After the birth [...] my midwife and surgeons were unsure of the severity of the tear, so I was taken to theatre. Again, staff were fantastic at explaining the procedure and talked through every step with me.”* (Between 30-34 years old, white, SVB, no epidural).  Another respondent reflected on the lack of information given to her during the examination and subsequent suturing:  *“After the birth I had several nurses and midwives look at my vagina to assess what stitching was needed as I had 2nd degree tears and I think it would have been helpful to provide a diagram of the vagina […] I didn’t feel like I had much knowledge or information about what was happening post birth but understood it was probably for the best. But I think a diagram would have helped me feel like I was making a better-informed decision about the consent I was providing. Other surgeries would involve this level of detail, so it feels disproportionate that birthing women aren’t given this.*” (Between 35-40 years old, white, SVB, no epidural) |
| --- |
